# Supplementary material for: FUS affects circular RNA expression in murine embryonic stem cell-derived motor neurons
Source: Nat Commun. 2017 Mar 30;8:14741. doi: 10.1038/ncomms14741 (PMC5379105; doi:10.1038/ncomms14741)
Supplement: Supplementary Information — Supplementary Figures, Supplementary Table and Supplementary References [file ncomms14741-s1.pdf]

Supplementary Figure 1

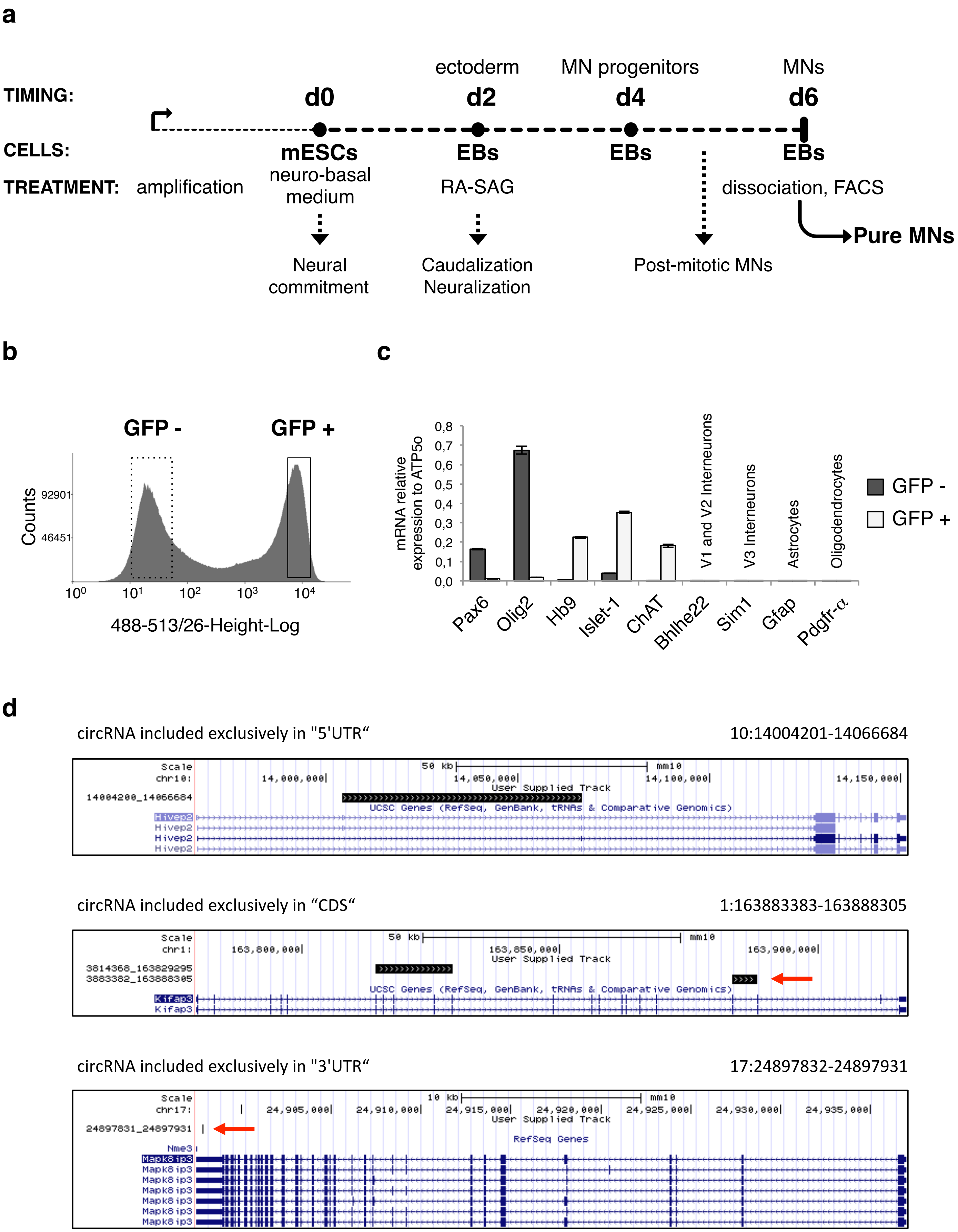

Supplementary Figure 1

circRNA included exclusively in “Intron“ 13:107840504-107840946

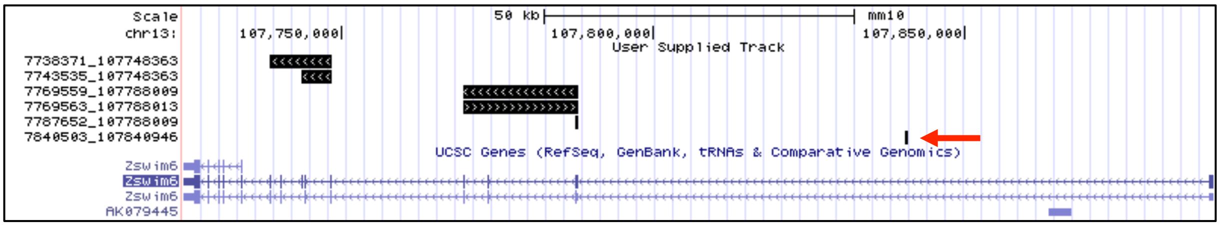

circRNA in "3'UTR" and “CDS” 14:104478607-104478809

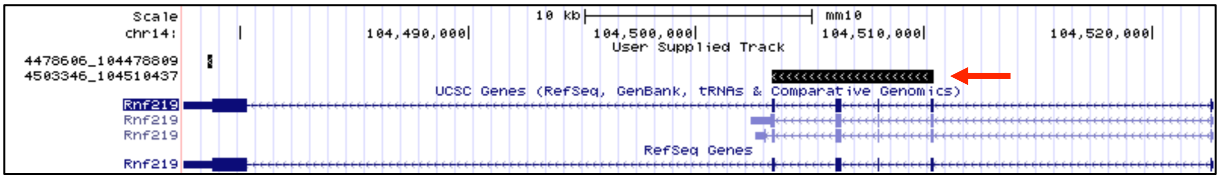

circRNA in "3'UTR", "5'UTR" and "cds“ 4:136540356-136545402

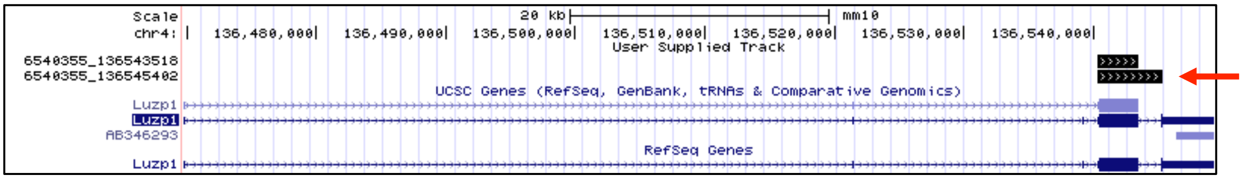

circRNA in "3'UTR", "5'UTR”, "cds" and "introns“ 19:4784724-4794009

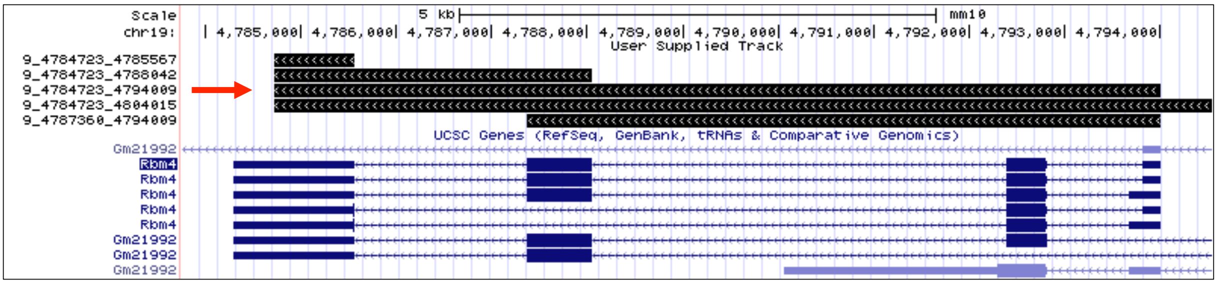

circRNA in "3'UTR", "cds" and "introns” 1:58409981-58414171

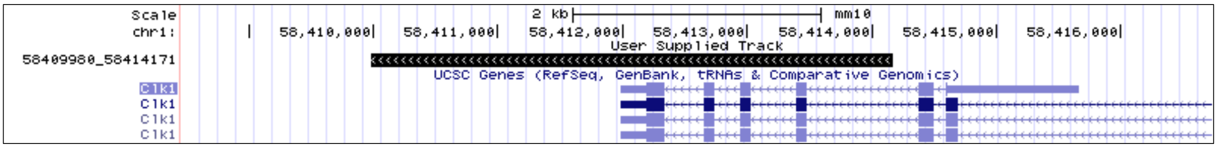

circRNA in "5'UTR" and "cds“ 5:143723895-143728026

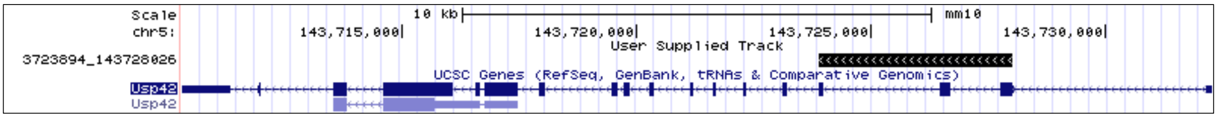

Supplementary Figure 1

circRNA in "5'UTR", "cds" and "introns“ 6:115637599-115669424

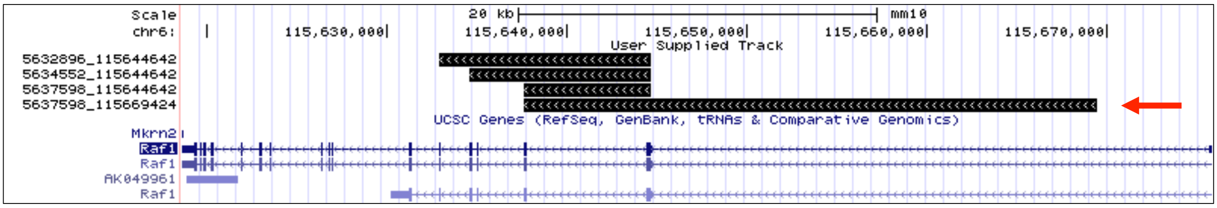

circRNA in "5'UTR" and "introns“ 1:97653130-97654689

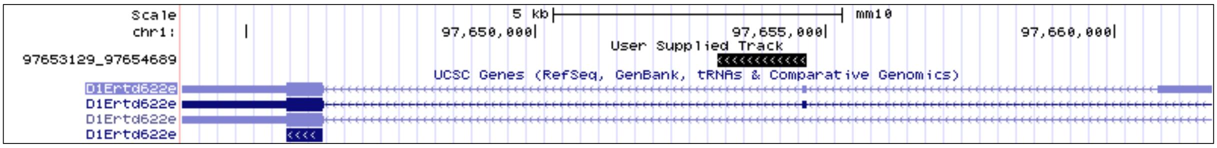

circRNA in "cds" and "introns“ 7:111597803-111599123

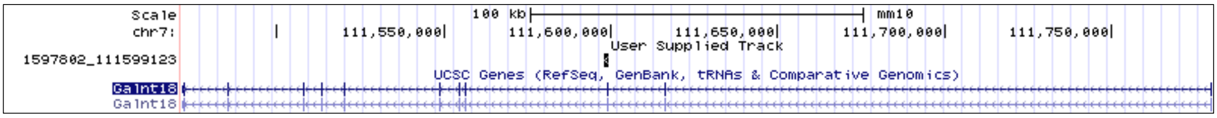

e

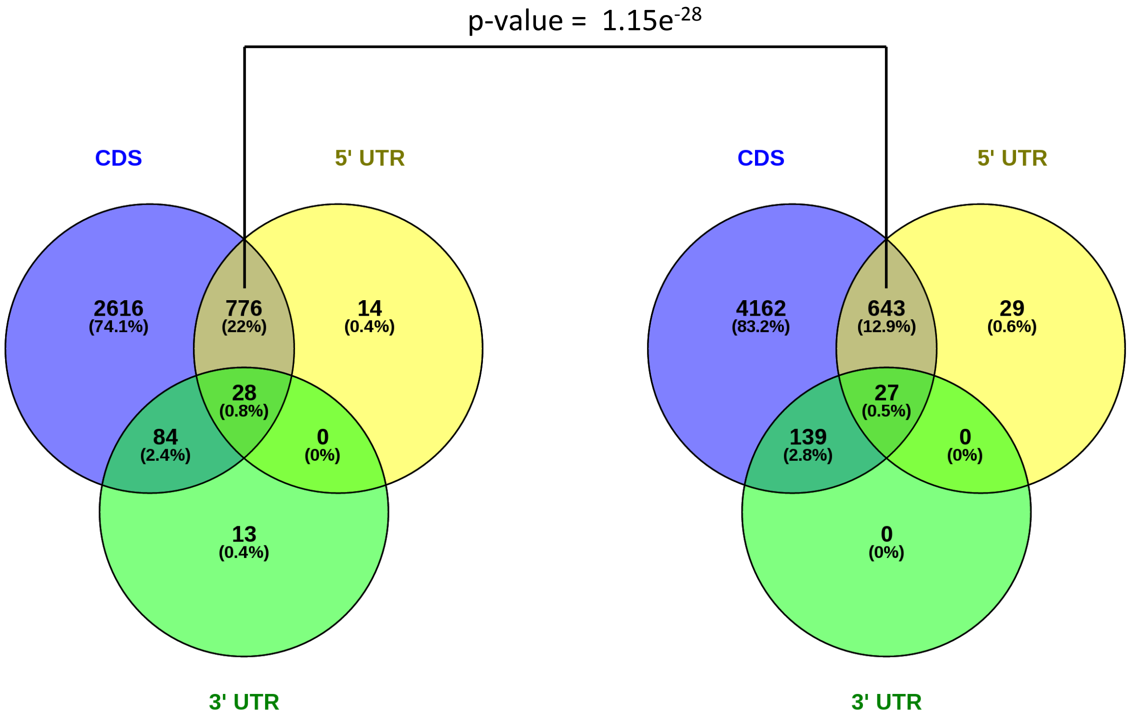

Motor neuron circRNAs **Faux random circRNAs**

Supplementary Figure 1

Supplementary Figure 1: (**a**) Mouse embryonic stem cells (mESCs), derived from wild type (*FUS*$^{+/+}$) or knock out (*FUS*$^{-/-}$)[1] FUS mice and expressing a GFP reporter under the control of the motor neuron-specific Hb9 promoter (*Hb9::GFP* transgene)[2], were differentiated into motor neurons according to Wichterle *et al*[3]. The procedure relies on the formation of embryoid bodies (EBs), neurally committed through maintenance in differentiation medium. Retinoic acid and smoothened agonist (RA-SAG) leads neural progenitors to caudal and ventral patterning respectively[2], which finally results into spinal motor neuron differentiation. After four days of RA-SAG treatment, EBs were dissociated and *Hb9::GFP+* cells were purified by FAC-sorting. (**b**) The distribution of the *Hb9::GFP-* (GFP-) and *Hb9::GFP*+ (GFP+) cell populations analysed by fluorescence-activated cell sorting (FACS); post-sorting cytofluorimetric evaluation highlighted a purity level higher than 98% of the indicated GFP+ fraction. (**c**) Histogram showing that the pool of *Hb9::GFP-* cells specifically express the Pax6 and Olig2 transcription factors that, within the domain of neuronal precursors, are responsible for establishing MN progenitors[2]. These markers are at almost undetectable levels in the GFP+ population. On the other hand, expression of genes required for consolidation of identity (*Hb9*), development (*Islet-1*) and function (*ChAT*) of spinal motor neurons was highly enriched in *Hb9::GFP+* cell fraction. As expected for the differentiation protocol utilized, the markers for astrocytes (*Gfap*) and oligodendrocytes (*Pdgfr-α*) were almost undetectable in both cell populations. Notably, also the V1 and V2 (*Bhlhe22*) and V3 interneuron (*Sim1*) markers were not detectable. These data confirm that the protocol adopted faithfully recapitulates *bona fide* motor neuron specification from mESCs, with motor neuron precursors mainly contained in the *Hb9::GFP-* cell pool, and the post-mitotic motor neurons present in the *Hb9::GFP+* cell fraction. (**d**) Genome browser plots for representative circRNAs from each category shown in Fig. 1C. (**e**) Localization of internal, non-intronic motoneuronal circRNAs and faux random circRNAs with respect to 5' UTR, 3'UTR and coding region of protein-coding transcripts. P-value was calculated performing Chi-square test comparing the number of real and faux circRNAs included in each region.

Supplementary Figure 2

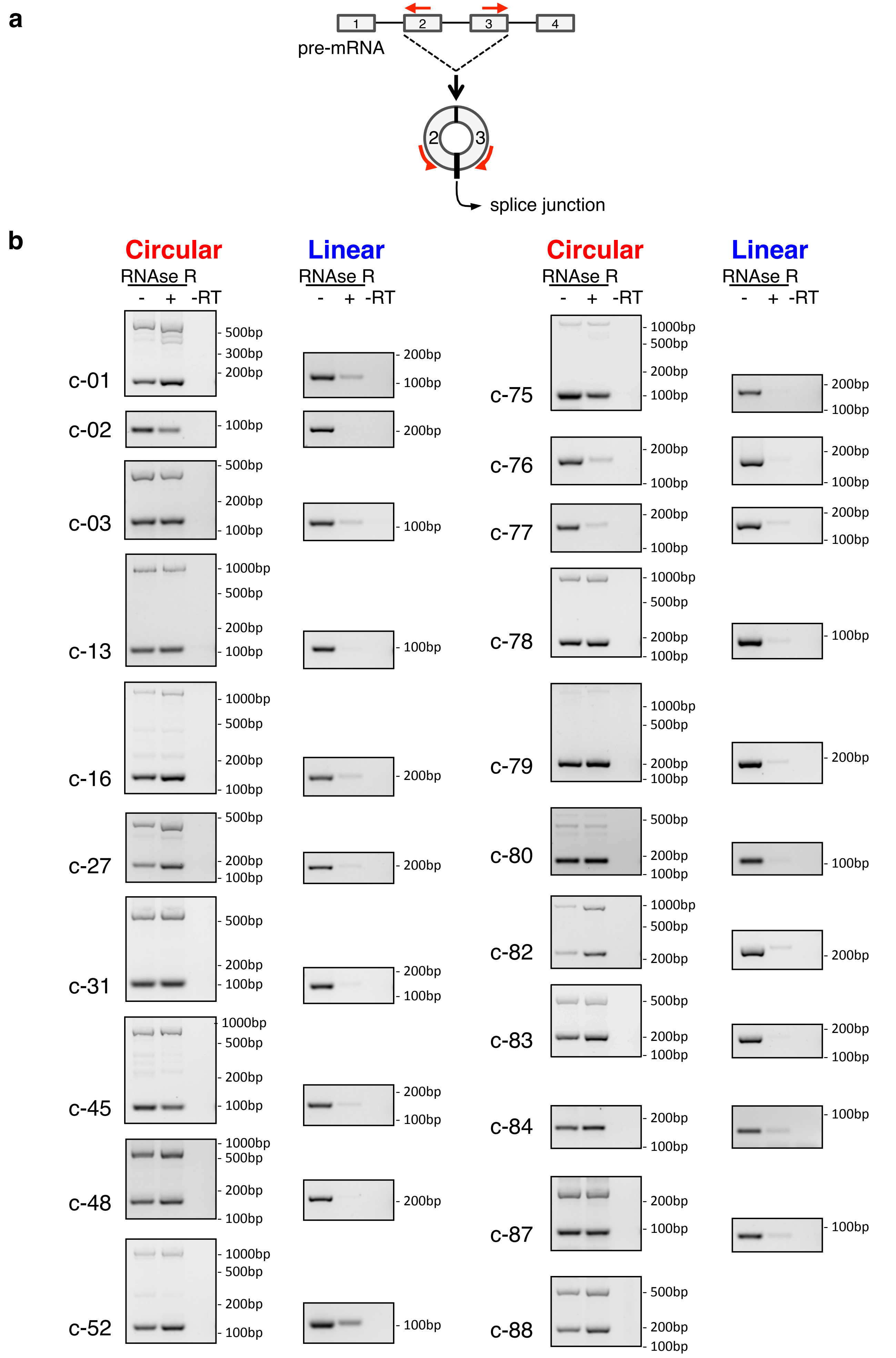

Supplementary Figure 2

c

c-01

exon 2
ATGCTTTCGTGAACAGCCAGGAATGGACACTCAGCCGATCGGTACCGGAGCTCAAAGTG
exon 3
GGAATTGTGGGTAACCTGGCCAGTGGCAAGTCTGCGCTGGTGCACCGGTACCTGACAGGCACGTACGTGCA
GGAGGAATCTCCAGAAG
exon 4
GTGGCAGGTTCAAGAAGGAGATTGTTGTTGATGGACAGAGTTATCTGCTGCTGATTAGGGATGAAGGGGGCC
CCCCGGAGGCACAG
exon 5
TTCGCCATGTGGGTGGACGCGGTCATCTTTGTCTTCAGCTTGGAGGATGAGATCAGTTTCCAGACTGTCTACC
ATTACTACAGCCGAATGGCCAACTACAGGAACACCAGTGAGATCCCATTAGTGCTGGTGGGGACCCAGG
exon 6
ATGCCATAAGTTCCACCAACCCGAGGGTCATCGATGACGTCAGGGCTCGGAAGCTCTCCAATGACCTGAAGA
GGTGCACGTACTATGAGACGTGCGCCACCTATGGGCTCAACGTGGAGCGGGTCTTCCAGGACG

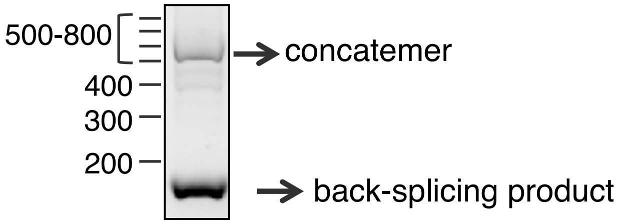

back-splicing product

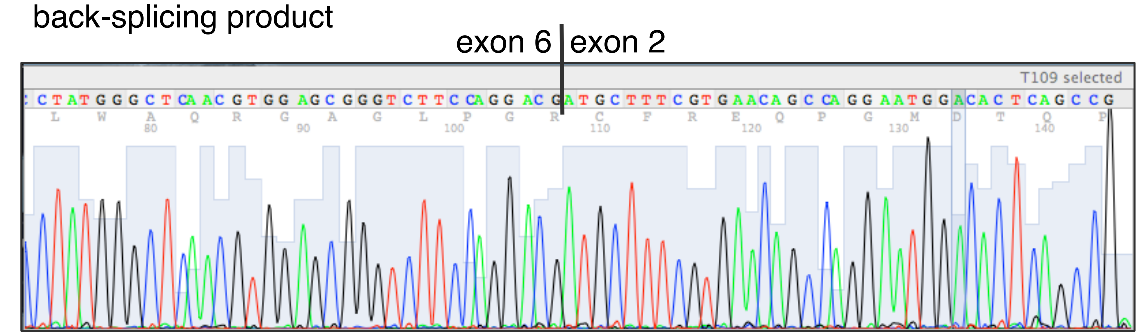

concatemer

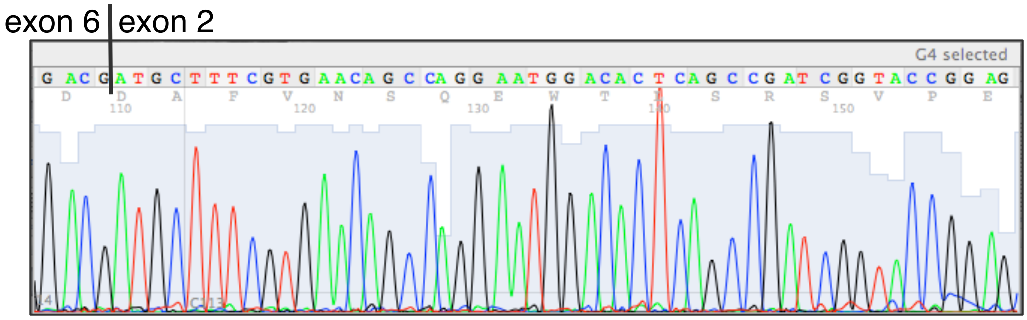

Supplementary Figure 2

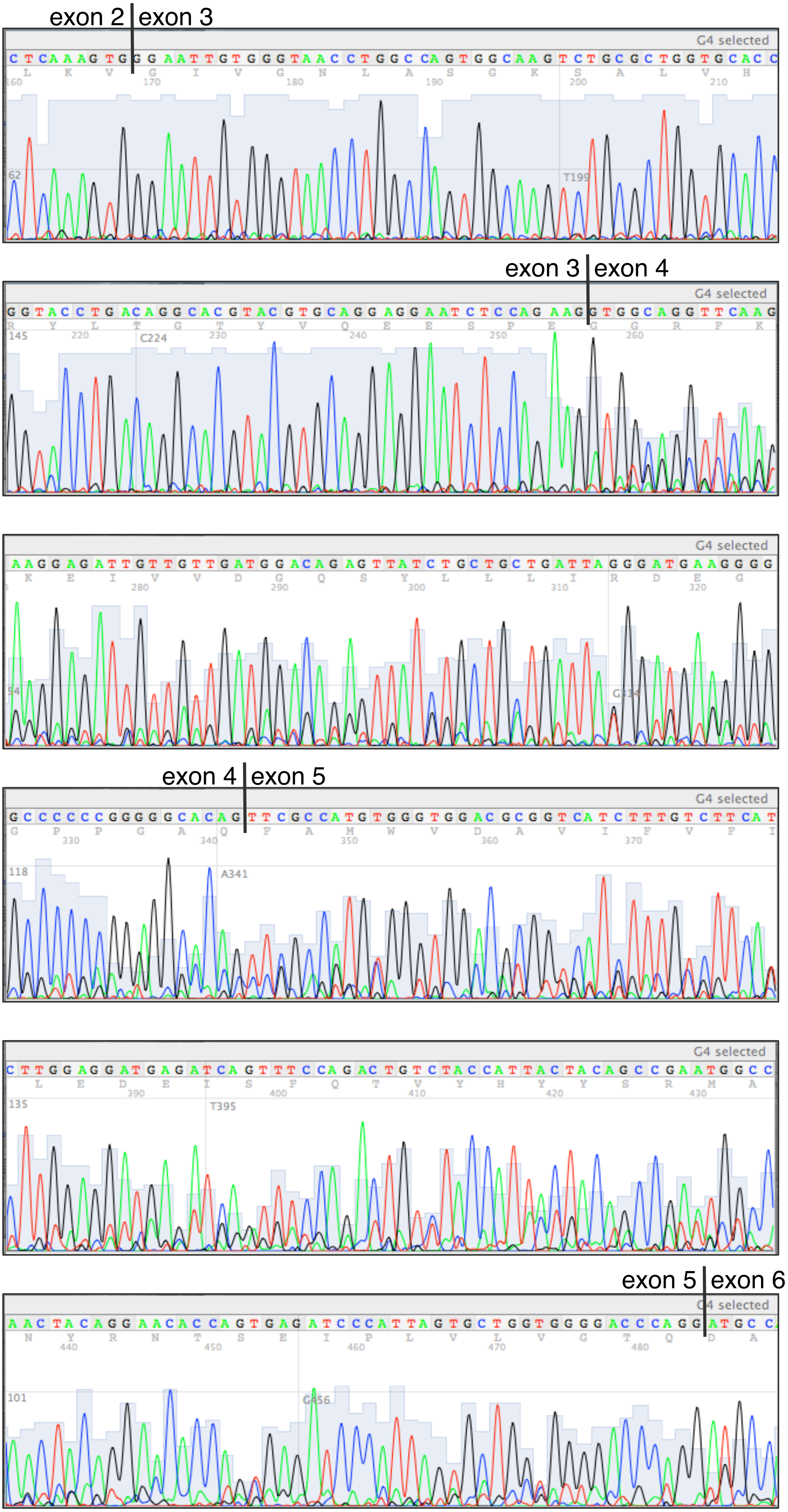

Supplementary Figure 2

d

c-87

exon 2
AATAATCATGGGCCAGACTGGGAAGAAATCTGAGAAGGGACCGGTTTGTTGGCGGAAGCGTGTAAAATCAGAG
TACATGAGACTGAGACAGCTCAAGAGGTTCAGAAGAGCTGATGAAGTAAAG

exon 3
ACTATGTTTAGTTCCAATCGTCAGAAAATTTTGGAAAGAACTGAAACCTTAAACCAAGAGTGGAAGCAGCGGAG
GATACAGCCTGTGCACATCATGACTTCT

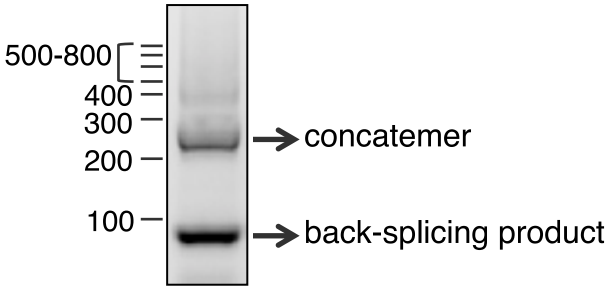

back-splicing product

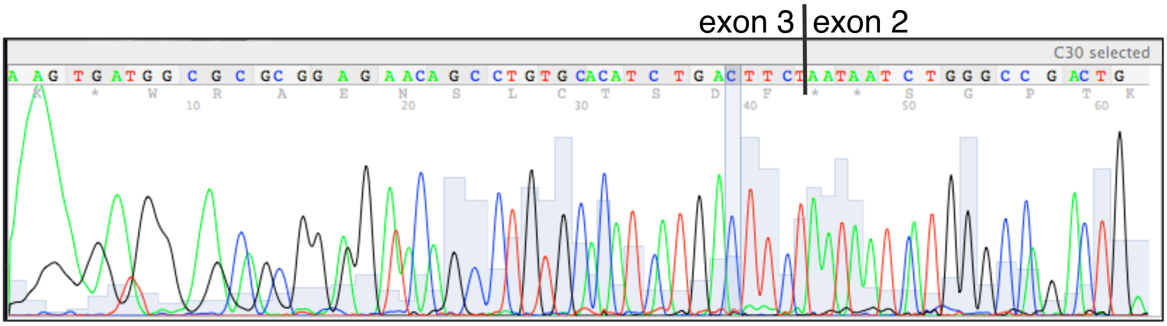

concatemer

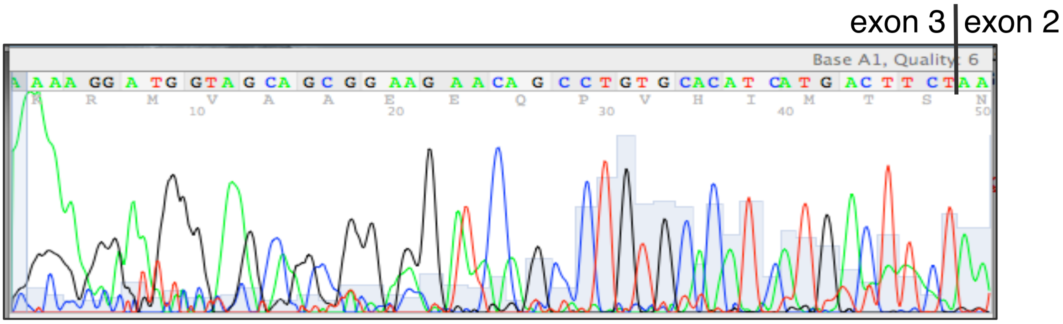

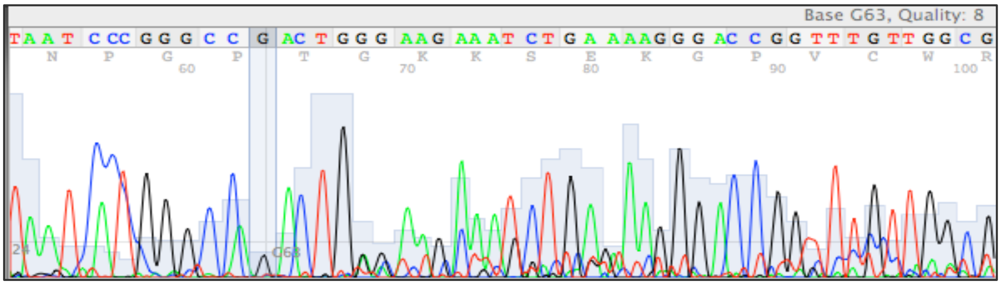

Supplementary Figure 2

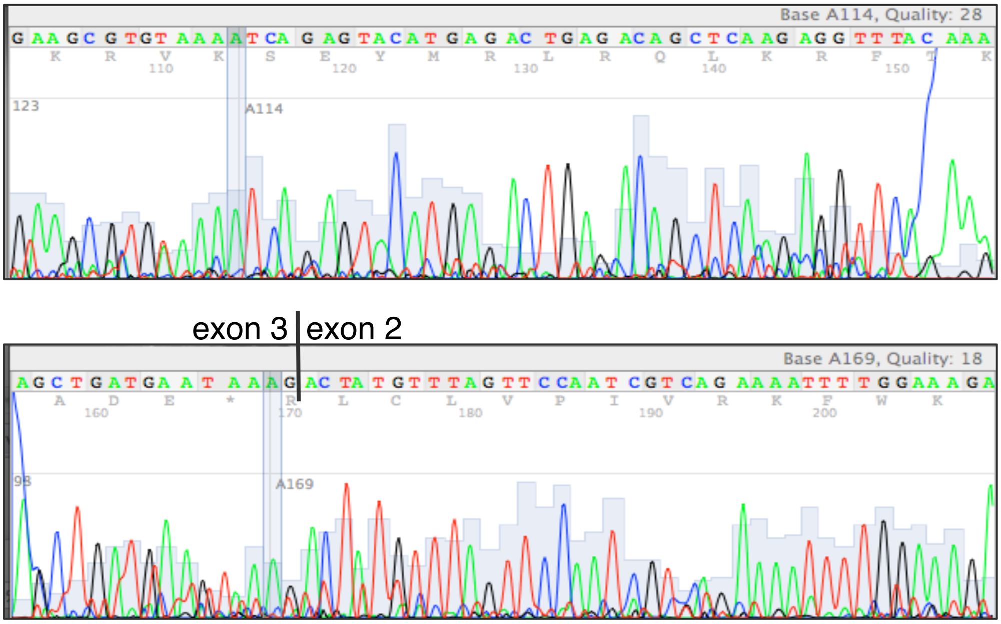

e

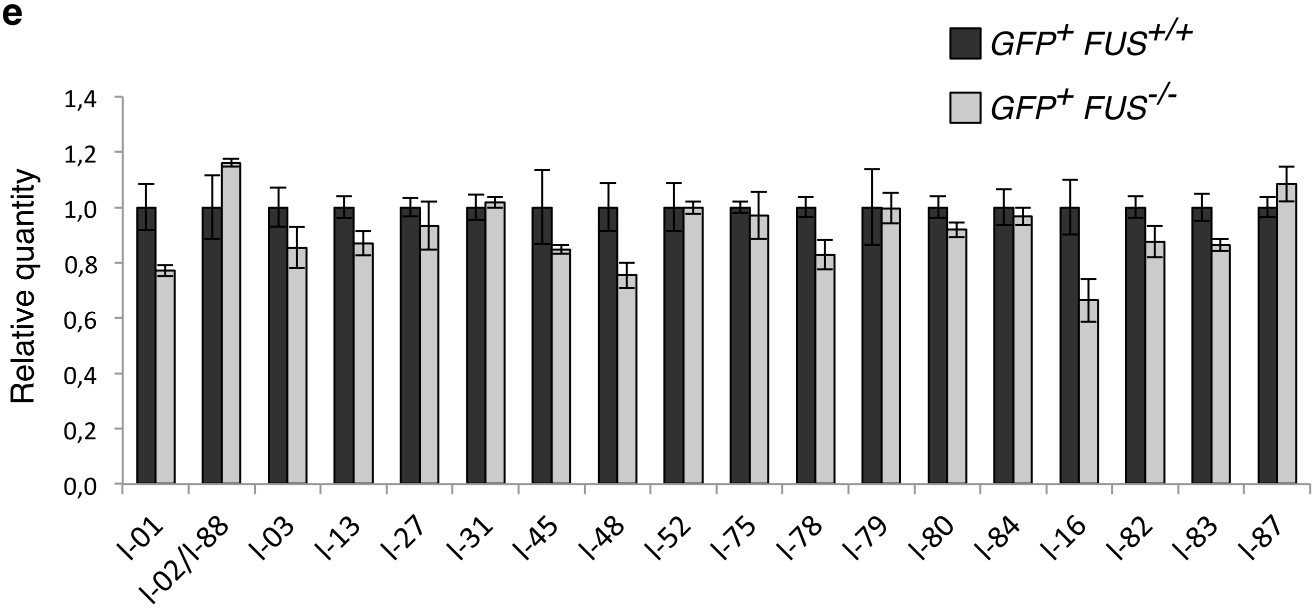

f

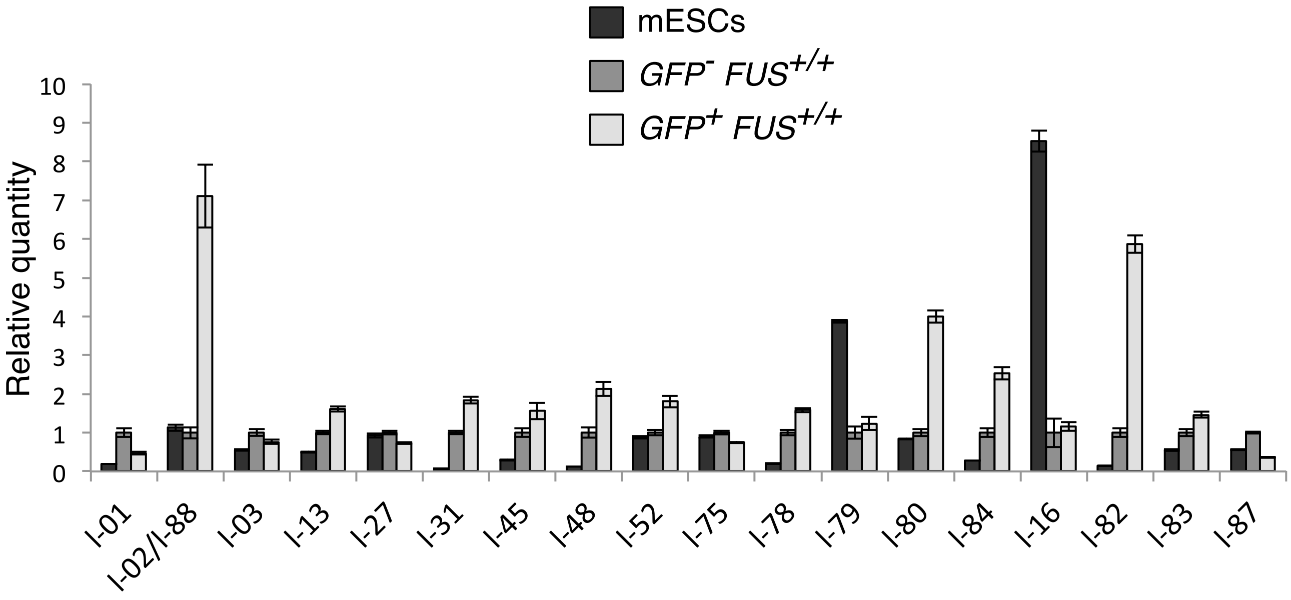

Supplementary Figure 2

g

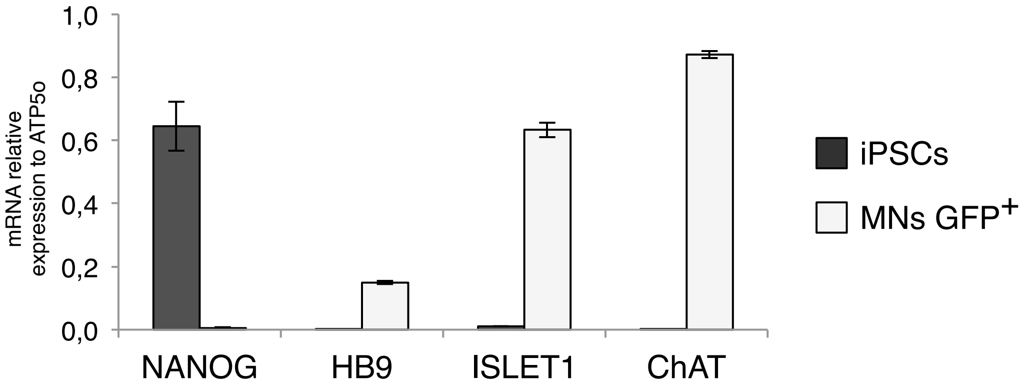

h

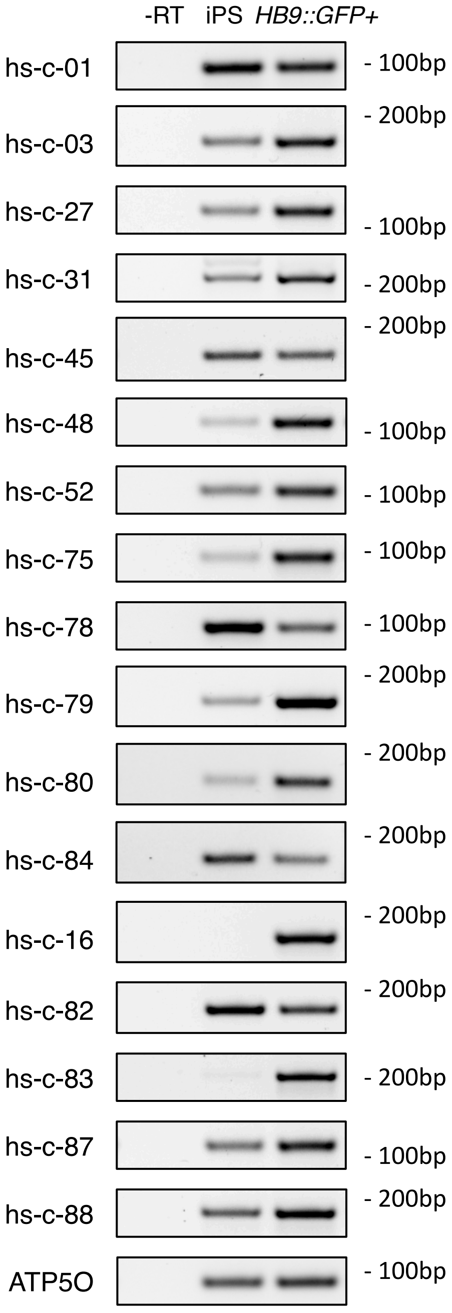

Supplementary Figure 2

Supplementary Figure 2:
(**a**) Schematic representation of the primers used for RT-PCR amplification of circular (red) and linear (blue) RNA molecules. (**b**) Total RNA extracted from EBs at d6 of differentiation was treated (+) or not (-) with RNAseR. For each circRNA molecule both the circular form and the linear counterparts are amplified through RT-PCR. Since c-02 and c-88 arise from the same gene, only one linear counterpart has been analysed and named l-02/l-88. (**c**) Upper panel: Sequence of the circularising exons for c-01; sequence of the forward oligonucleotide used for sequencing is underlined; middle panel: agarose gel showing the results of RT-PCR on EBs using divergent oligonucleotides listed in Supplementary Table 3. Lower panels: electropherograms showing the sequence of both back-splicing product and concatemer detected in RT-PCR analysis. The linear and back splice junctions are indicated. (**d**) Upper panel: sequence of the circularising exons for c-87; sequence of the forward oligonucleotide used for sequencing is underlined; middle panel: agarose gel showing the results of RT-PCR on EBs using divergent oligonucleotides listed in Supplementary Table 3. Lower panels: electropherograms showing the sequence of both back-splicing product and concatemer detected in RT-PCR analysis. The linear and back splice junctions are indicate. (**e**) Histograms show the expression level of linear host transcripts for circRNAs analysed in Fig. 2a measured by qRT-PCR in sorted *GFP*$^{+}$*-FUS*$^{+/+}$ and *GFP*$^{+}$*-FUS*$^{-/-}$ cells. The linear RNA levels were normalized toward Atp5o mRNA levels and expressed as relative quantity respect to *GFP*$^{+}$*-FUS*$^{+/+}$ sample set to a value of 1. Error bars represent s.e.m. of three independent experiments and two-tailed Student's t-test was applied. (**f**) The histogram shows the expression level of linear transcripts shown in Fig. 2b, measured by qRT-PCR, in FUS$^{+/+}$ mESCs, GFP^{+} and GFP^{-} cells. The linear RNA levels were normalized toward Atp5o mRNA levels and and expressed as relative quantity respect to GFP^{-} samples set to a value of 1. Error bars represent s.e.m. of three independent experiments. (**g**) Analysis of MN markers in iPSCs and in human *HB9::GFP+* cells (MNs GFP+). mRNA levels are normalized against Atp5o mRNA levels. (**h**) *HB9::GFP* human iPSCs were either maintained in proliferation medium or induced to MN differentiation and the GFP^{+} ^{f}raction purified by FAC sorting. CircRNA expression was analysed by RT-PCR and ATP5O mRNA was used as endogenous control. –RT samples were also analysed as control.

Supplementary Figure 3

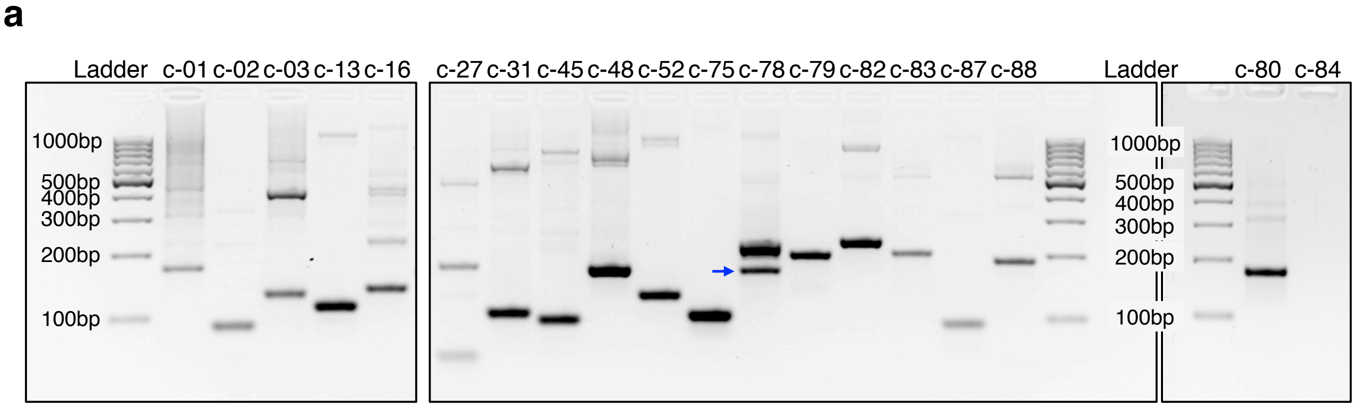

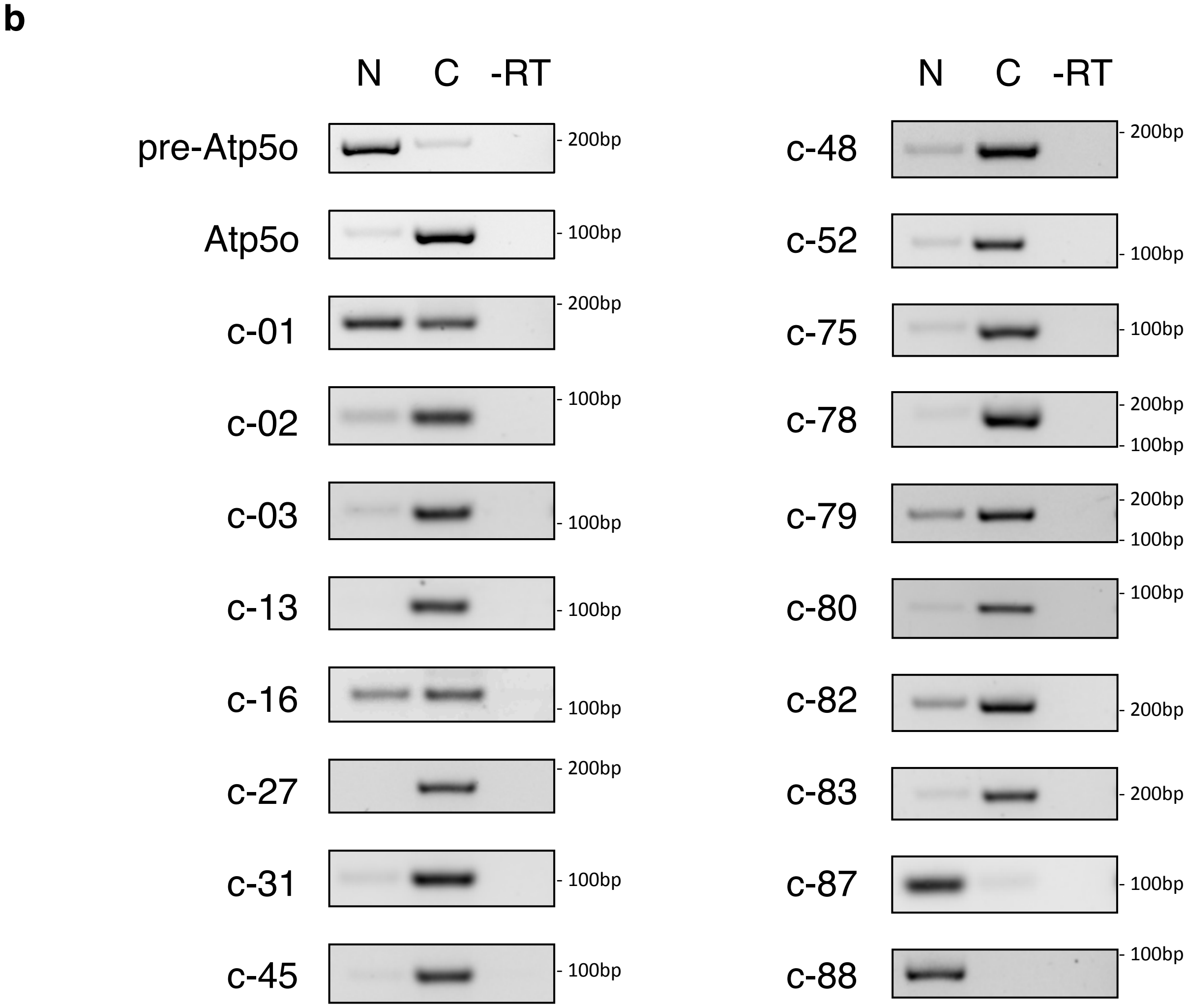

Supplementary Figure 3

c

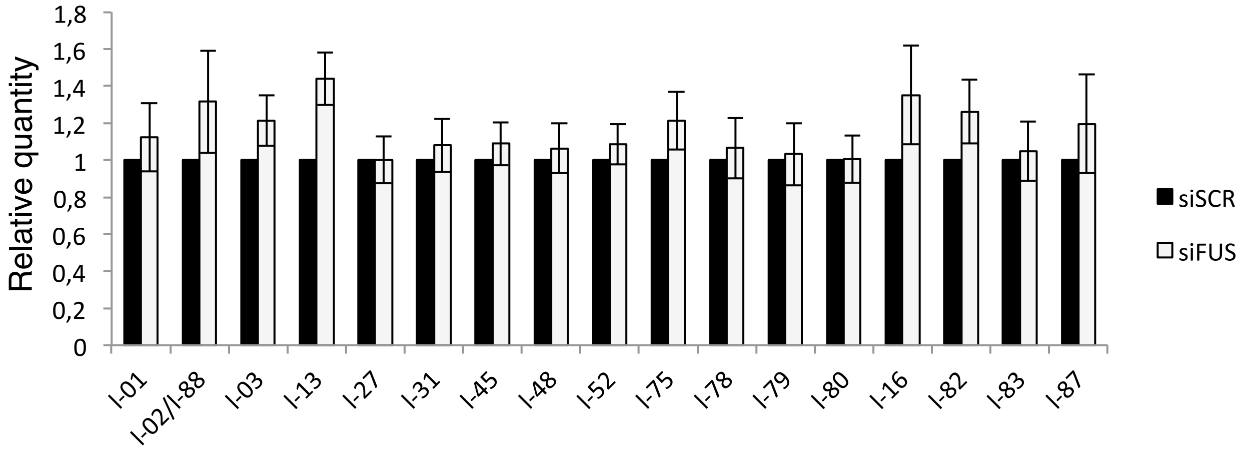

d

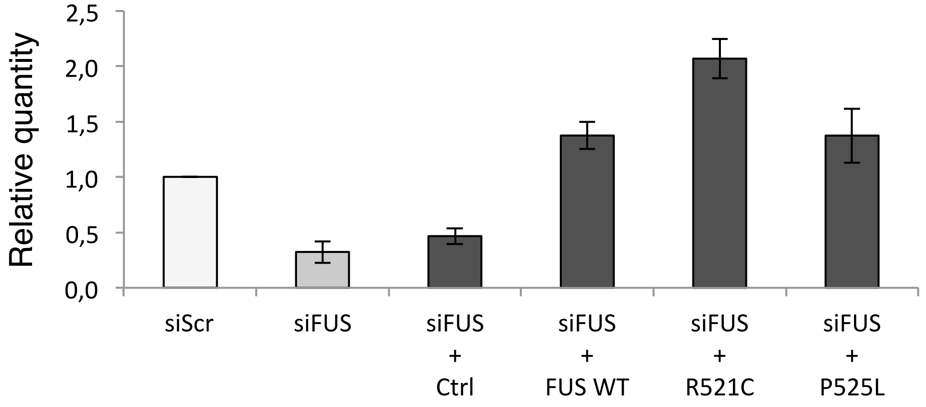

Supplementary Figure 3:
(**a**) The expression of 19 circRNAs in differentiated N2a cells assayed through RT-PCR. The arrow indicates the specific amplification product for c-78. (**b**) The cellular sub-localization of selected circRNAs was analysed in nuclear (N) and cytoplasmic (C) compartments in differentiated N2a cells. The RT-PCR analyses were performed using specific primers for the circRNA molecules, as schematically represented in Supplementary Fig. 2a. Pre-Atp5o and Atp5o transcripts are used as control to check the quality of nuclear/cytoplasmic fractionation. (**c**) Histograms show the expression levels of the linear RNA counterparts of the circRNAs analysed in Fig. 3b, in differentiated N2a cells depleted (siFUS) or not (siScr) for FUS, analysed by qRT-PCR. circRNAs levels were normalized toward Atp5o mRNA and the values were then normalized to siScr samples set to a value of 1. Since c-02 and c-88 arise from the same gene, only one linear counterpart has been analysed and named l-02/ l-88. Error bars represent s.e.m. of three independent experiments. Error bars represent s.e.m. of three independent experiments and two-tailed Student's t-test was applied. (**d**) Histograms show the quantification of four western blot analyses of the experiments in Figure 3a, 3b and 3c. The values were normalised for GAPDH expression. Error bars represent s.e.m. of four independent experiments

Supplementary Figure 4

a

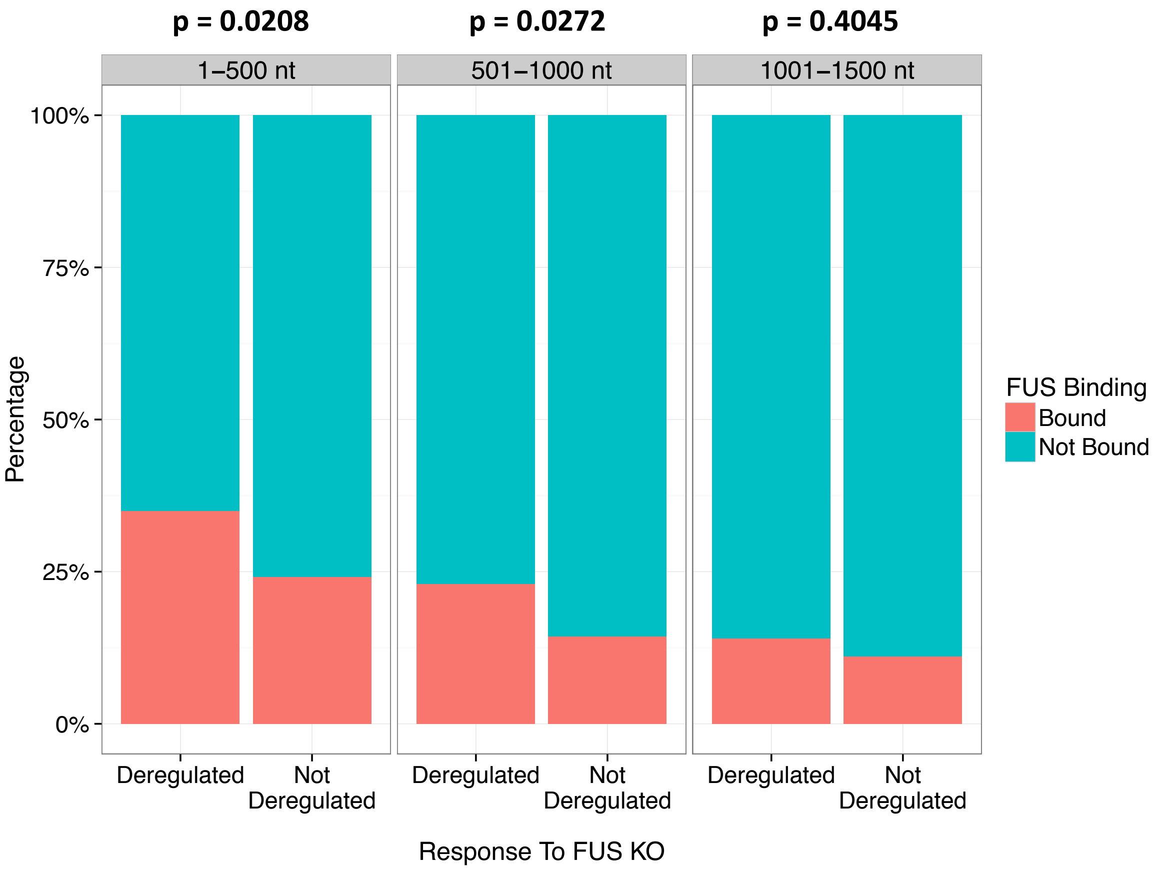

b

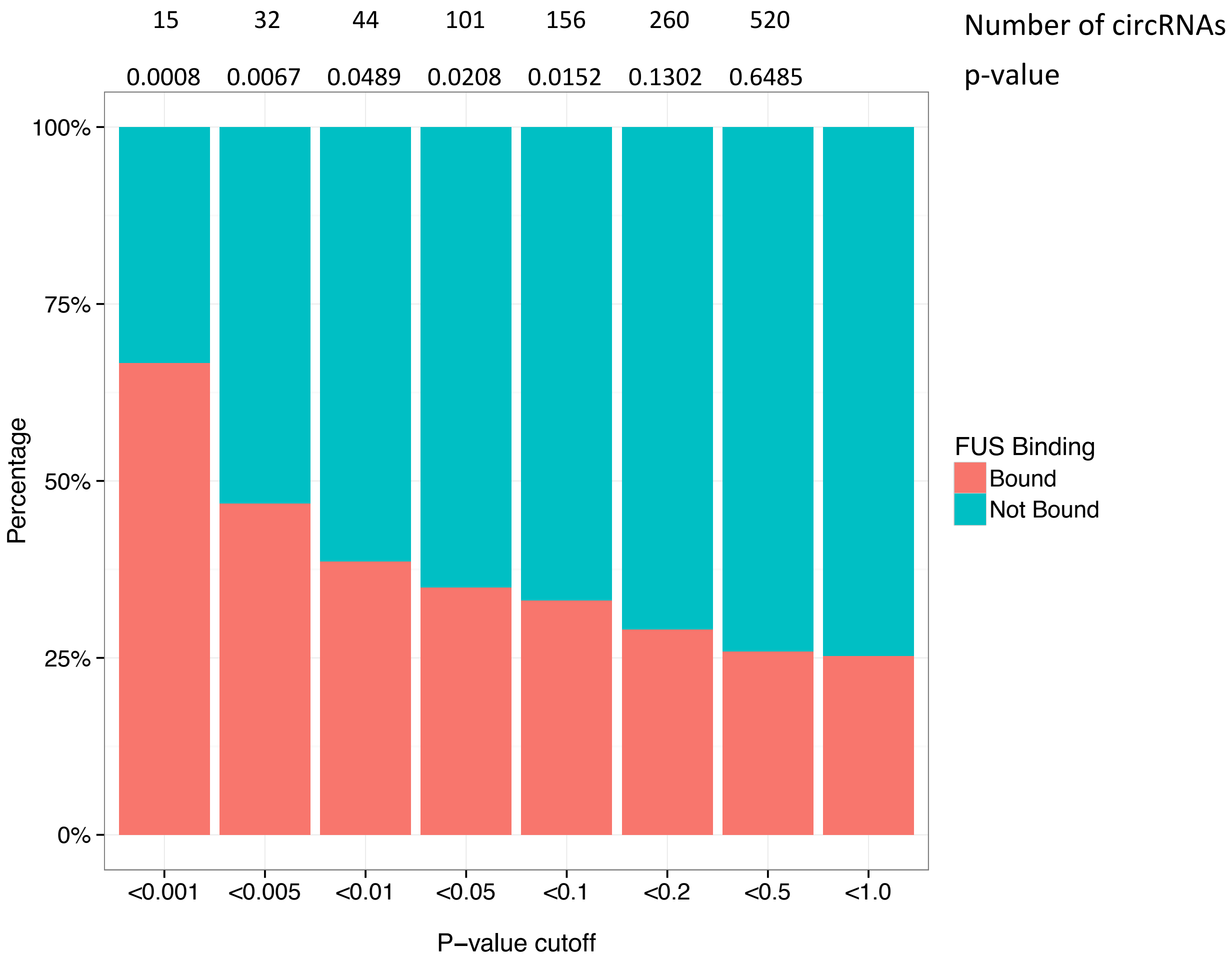

Supplementary Figure 4

c

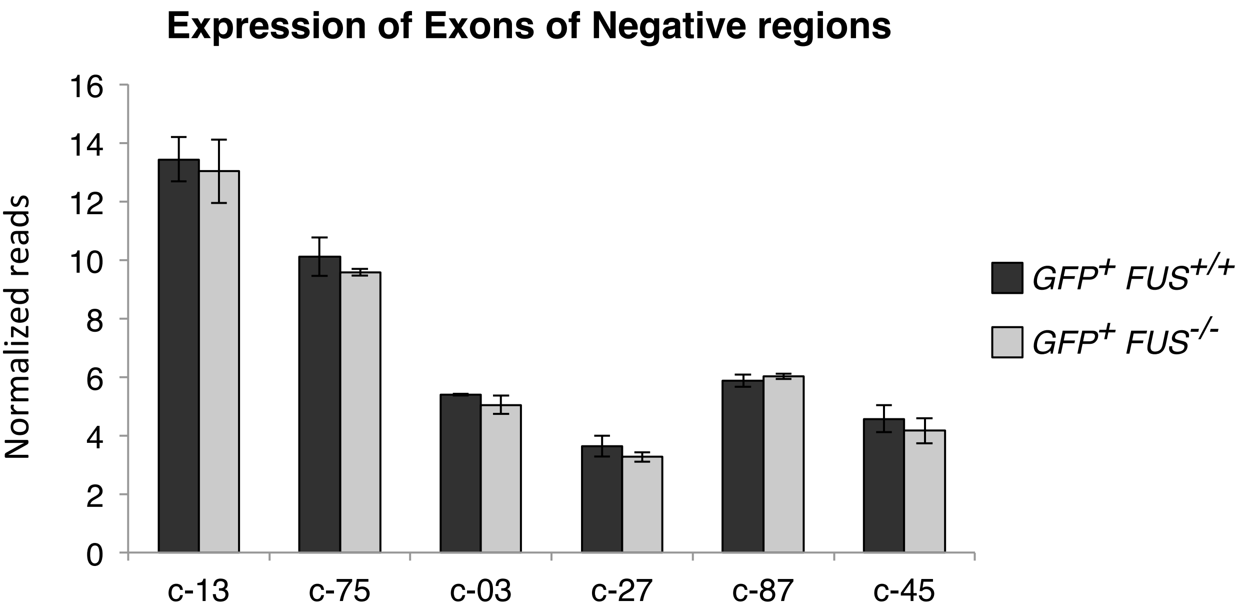

Supplementary Figure 4:

(**a**) Bar plot showing the fraction of deregulated and unaltered circRNAs whose flanking intronic regions (divided into three 500 nt-long regions) are bound by FUS. (**b**) Bar plot showing the fraction of deregulated circRNAs (selected using different p-value thresholds) whose flanking 500 nt-long intronic regions are bound by FUS. (**c**) Histogram shows the quantification of the expression of the exons used as negative loci in CLIP experiments (NEG, Fig. 4d) in *FUS*$^{+/+}$ and *FUS*$^{-/-}$ *GFP*$^{+}$ samples. The data shown arise from RNAseq analysis and are calculated as number of reads normalized on millions of reads of each sample.

Supplementary Figure 5

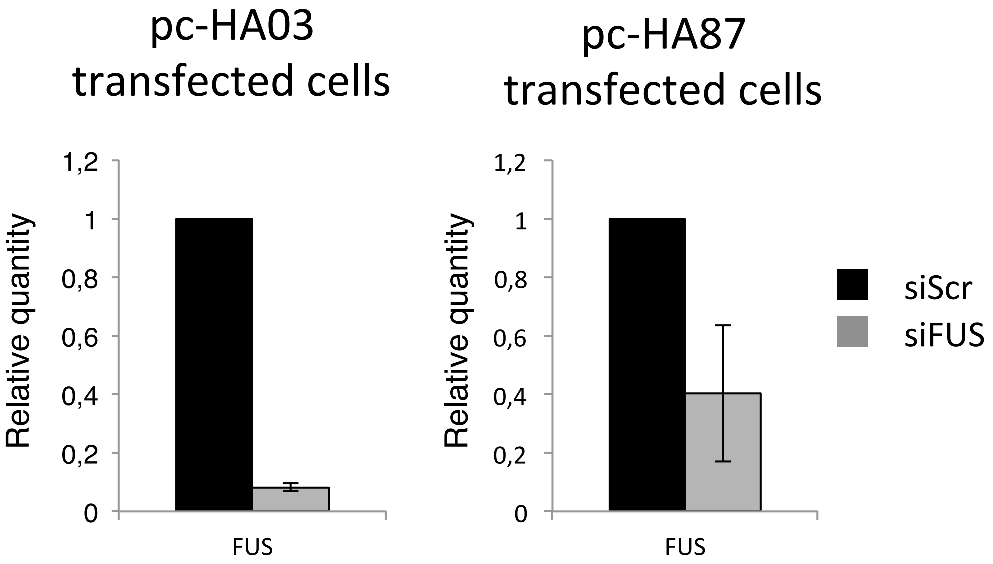

Supplementary Figure 5:
FUS RNA levels in N2a cells depleted (siFUS) or not (siScr) for FUS and transfected with pc-HA03/87 constructs.

Supplementary Figure 6

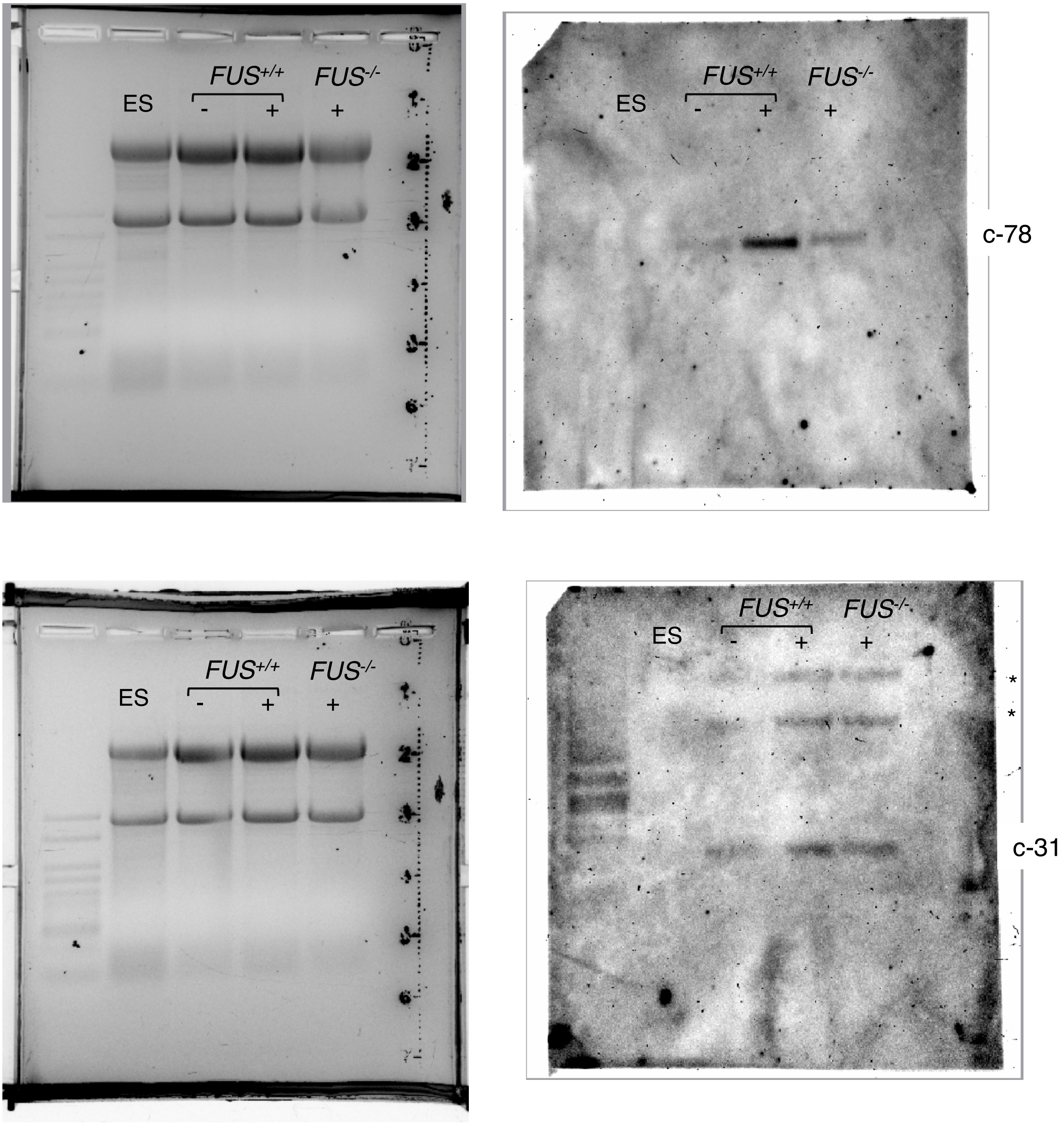

Supplementary Figure 6:
Uncropped versions of Northern blots shown in Supplementary Figure 2c.

Supplementary Figure 7

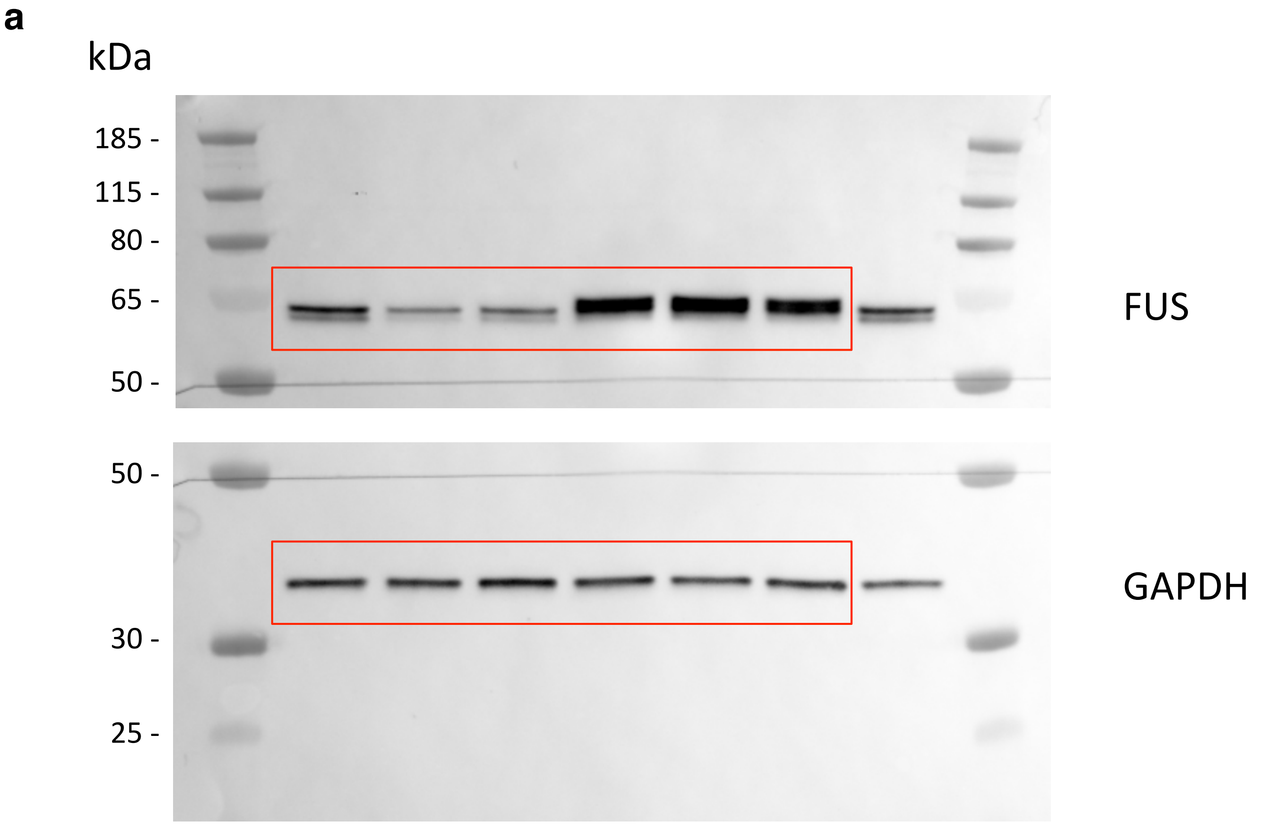

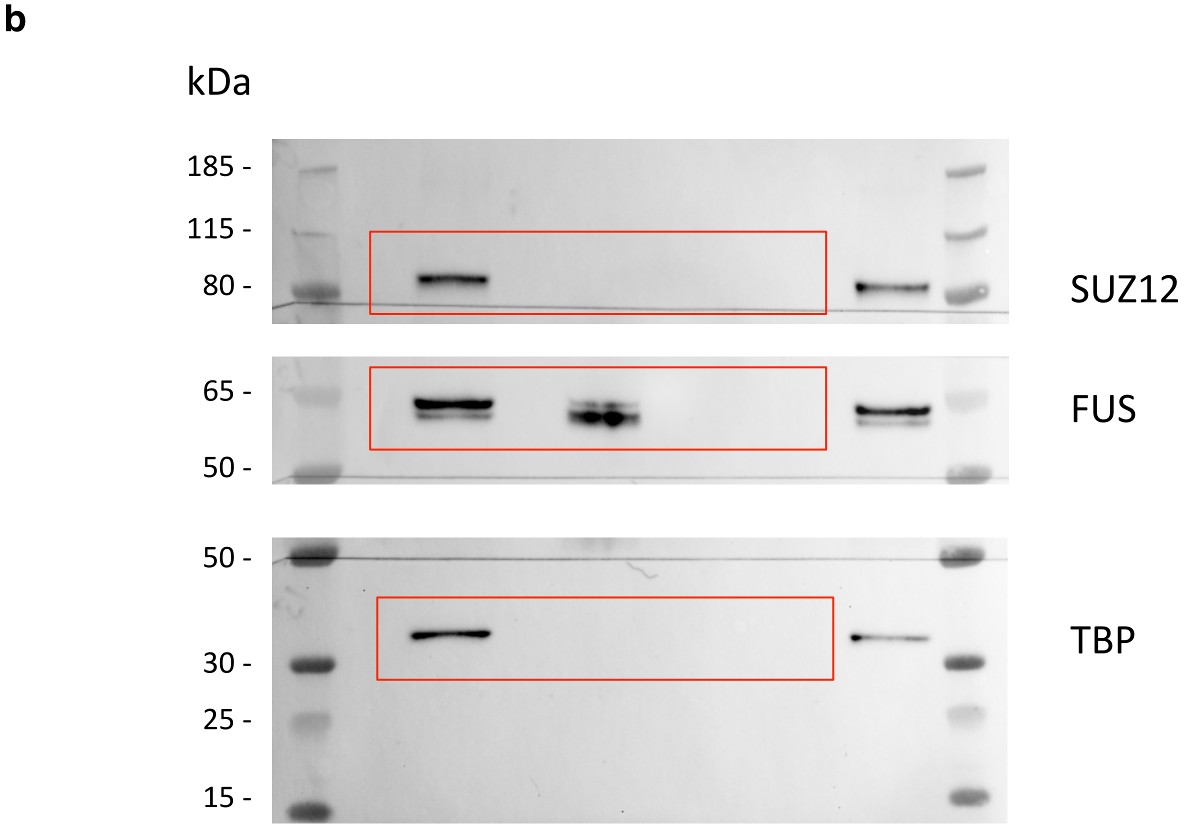

Supplementary Figure 7:
(**a**) Uncropped versions of Western blots shown in Figure 3a. (**b**) Uncropped versions of Western blots shown in Figure 4a.

Supplementary References

1 Hicks, G. G. *et al.* Fus deficiency in mice results in defective B-lymphocyte development and activation, high levels of chromosomal instability and perinatal death. *Nat. Genet.* **24,** 175–9 (2000).

2 Wichterle, H., Lieberam, I., Porter, J. A. & Jessell, T. M. Directed differentiation of embryonic stem cells into motor neurons. *Cell* **110,** 385–397 (2002).

3 Wichterle, H., Peljto, M., Wichterle, H. & Peljto, M. Differentiation of Mouse Embryonic Stem Cells to Spinal Motor Neurons. *Curr. Protoc. Stem Cell Biol.* **Chapter 1,** 1H.1.1–1H.1.9 (2008).

Supplementary Table 1. List of oligonucleotides used for the experiments.

The sequence of the oligonucleotides are indicated in 5'-3' direction.

	Forward	Reverse
**Primers used for RT-PCR analysis in mouse samples:**		
c-01	ATGCCATAAGTTCCACCAACCC	CGGCTGAGTGTCCATTCCTG
c-02	ACTTCCCCTTGCTGAGCTTG	TACTGTGAACCTGGGCTCGG
c-03	CAAGCTGGACCGCAGTGATG	GCTATTGCTTCATCATCAACTGTCTGT
c-13	ATCCATGCTTTGCCCGAGAG	CCCAAACCCAATTTCTGACTGT
c-16	CGTGACCACCCAGGAGACTG	ATATGACACTCAAAGGGATGGAAGT
c-27	GCAGCAGCTTGTCAATTCAGTTAT	TCCAAAAAGATGTGTCACAAGCC
c-31	AGACGCTGGCAATGACACGA	TACTTGTTTGCTGGAGGCTTCA
c-45	GGAGACACTATGGAGAATGTGGAAG	CCTCATCCTCTTCTTCATCAGTGG
c-48	AGAATGACGGCAGCCAGTCC	GGCGATTTCTCTCCTCTATATCCAT
c-52	CTGACAGGAGCAACAGAGGGTG	CCTCCTCCTGTGACACTCTTGATAC
c-75	CTCACAGGTAGATTCTGGCACG	GCATACTCTAACATCTTTATTCTTCTCAC
c-76	GAAGATCCACTACTGTCTCCTGTTG	CTTTGTAGTTCCCTCATTGCCA
c-77	ACCTTCAACCCACCAACACG	CTTTGTAGTTCCCTCATTGCCA
c-78	TCGTATGGGGCAGTTTAGGACT	CACGCCTTCATAGTAAAGCTGG
c-79	CACGTTCTCTGAACCAGAACCG	TTCCAAGCCCAGATCCTGTG
c-80	GCAGGGGCTCACAGGAAACAT	GCATTTCAAACCATCTCCATCTTC
c-82	CCAACTCAAGGCATGTCTGTGT	GCTTCCCTCAGCTCGGTGAA
c-83	TTGTCCAAAAGAAGTCACAGATCAA	ATTCAAGCATTGGAAGAATTTAGAG
c-84	CGGGAGAAATGGATCTACTACC	TTCCTGCTGGGCACTTGTCG
c-87	CAGTCTGGCCCATGATTATTAGAAGT	TTGGAAAGAACTGAAACCTTAAACC
c-88	ATACAAGGGTTTACAGATTGGGAC	GGCTCAGTATCTTAGGCAAAGTCTAG
l-01	GTCCACCCCAACATCTACTCCA	CGGCTGAGTGTCCATTCCTG
l-02/l-88	ATACAAGGGTTTACAGATTGGGAC	TCAACATCTAACTCATCATTGCTTTC
l-03	CCCTGCTGGAGTTCATACCC	CACCCTTGGGCATCGTTCTC
l-13	ATCAGCAGCCCACCCTTTGA	CTTTTCCAATTTGATGTAGGTTTCC
l-16	CGTGACCTCAGCCAGCAATG	ATATGACACTCAAAGGGATGGAAGT
l-27	CTTCACCCACCCTTCCTTCG	CGCTCATCCTCACTCCAGAAA
l-31	GCGAGCAGGAGAACGACCG	TACTTGTTTGCTGGAGGCTTCA
l-45	GGAGACACTATGGAGAATGTGGAAG	AGAGCCGTGTCCTTGTGGG
l-48	AGAATGACGGCAGCCAGTCC	TCATCCAGTTCCTCATCTCCACT
l-52	AGACTCATCGCTCGCCTTCC	ATTACTGCCAAGTTGTGCTCCG
l-75	ATCCTACACTACATCCAGCACGA	GCATACTCTAACATCTTTATTCTTCTCAC
l-76	GAAGATCCACTACTGTCTCCTGTTG	TTACTTTCCCAATTTCTACGTGTTG
l-77	ACCTTCAACCCACCAACACG	CCACTAACACGGTATAGTCCTTCAG
l-78	CATTGAATATAGACCTGAAAACCATC	CCTCTTTCCATCGCTAAGTGC
l-79	GCCTTCAGCCTCAGAACCG	TTCCAAGCCCAGATCCTGTG
l-80	AGGCAGAGGCAAGAGCAACT	GCATTTCAAACCATCTCCATCTTC
l-82	GCTTCCCTCAGCTCGGTGAA	CACTTGTAAGGCTTCTCACCTGTGT
l-83	CTCTATCTGTAGGTGCTGCTGCC	CCTCCATCGAGAAAATAATCCAC
l-84	CGGCTCTCTTCTCCTGCTTGG	AGAGTCTTTTGTTCTGGTTGAGC
l-87	TTGGAAAGAACTGAAACCTTAAACC	TCACTGGTGACTGAACAAGAAGTC
Atp5o	CAACCGCCCTGTACTCTGCT	GGATTCAGAACAGCCAGAGACAC
pre-ATP5o	TGGAACTTACAGCAACACACGC	CTCAGTGACAATGCCATCCCTA
**Primers used for RT-qPCR analysis in mouse samples:**		
c-01	ATGCCATAAGTTCCACCAACCC	TGGCTGTTCACGAAAGCATCG
c-02	ACTTCCCCTTGCTGAGCTTG	TACTGTGAACCTGGGCTCGG
c-03	CAAGCTGGACCGCAGTGATG	GCTATTGCTTCATCATCAACTGTCTGT
c-13	AAACTCTTCTTATCCAGAGACAGTCAG	TGTACCCTCTCCGAGCTTTTCC
c-16	AAGCACTCTCGGGGTCAGCA	GATCTGCCATGTTGTCCACTTTC
c-27	GAGATCAACTTCCGTGGAGAATG	CCTTTACGCCAGCAAATAACTGA
c-31	AGACGCTGGCAATGACACGA	TACTTGTTTGCTGGAGGCTTCA
c-45	GGAGACACTATGGAGAATGTGGAAG	CCTCATCCTCTTCTTCATCAGTGG
c-48	AGAATGACGGCAGCCAGTCC	GGCGATTTCTCTCCTCTATATCCAT
c-52	CTGACAGGAGCAACAGAGGGTG	CCTCCTCCTGTGACACTCTTGATAC
c-75	CTCACAGGTAGATTCTGGCACG	GCATACTCTAACATCTTTATTCTTCTCAC
c-78	TCGTATGGGGCAGTTTAGGACT	CACGCCTTCATAGTAAAGCTGG
c-79	CACGTTCTCTGAACCAGAACCG	AAGATGATTTTCACAATAAACAGCAG
c-80	GCAGGGGCTCACAGGAAACAT	GCATTTCAAACCATCTCCATCTTC
c-82	CCACCAGAGGACTCACACAGACAT	GAAGCAGTCCAAGTCCTCACCA
c-83	TTGTCCAAAAGAAGTCACAGATCAA	TGTGTACTTGTGGTATCTTCCTGATG
c-84	CGGGAGAAATGGATCTACTACC	TTCCTGCTGGGCACTTGTCG
c-87	AAGACTATGTTTAGTTCCAATCGTCAG	CAGTCTGGCCCATGATTATTAGAAGT
c-88	ATCTGCCCTCCAACCTGCTT	GGCTCAGTATCTTAGGCAAAGTCTAG
l-01	GTCCACCCCAACATCTACTCCA	CGGCTGAGTGTCCATTCCTG
l-02/l-88	ATCTGCCCTCCAACCTGCTT	TCAACATCTAACTCATCATTGCTTTC

l-03	CCCTGCTGGAGTTCATACCC	CACCCTTGGGCATCGTTCTC
l-13	ATCAGCAGCCCACCCTTTGA	TGTACCCTCTCCGAGCTTTTCC
l-16	CGTGACCTCAGCCAGCAATG	ATATGACACTCAAAGGGATGGAAGT
l-27	GAGATCAACTTCCGTGGAGAATG	CGCTCATCCTCACTCCAGAAA
l-31	GCGAGCAGGAGAACGACCG	TACTTGTTTGCTGGAGGCTTCA
l-45	GGAGACACTATGGAGAATGTGGAAG	AGAGCCGTGTCCTTGTGGG
l-48	AGAATGACGGCAGCCAGTCC	CGTGCTTCTCTGAACAGTGCTCC
l-52	AGACTCATCGCTCGCCTTCC	ATTACTGCCAAGTTGTGCTCCG
l-75	CGCAACAGTACACGATCCCG	AAAATGCAATCCGTGCCTGC
l-78	ATAGACCTGAAAACCATCTTCTTTATG	GCCAGGTGTTCTTCAAAACAATG
l-79	GCCTTCAGCCTCAGAACCG	AAGATGATTTTCACAATAAACAGCAG
l-80	AGGCAGAGGCAAGAGCAACT	GCATTTCAAACCATCTCCATCTTC
l-82	GCTTCCCTCAGCTCGGTGAA	CACTTGTAAGGCTTCTCACCTGTGT
l-83	CCTGCTCCAGAGGCTCTACGC	TGAGTTGTCCAAAAGAAGTCACAGCT
l-84	CGGCTCTCTTCTCCTGCTTGG	AGAGTCTTTTGTTCTGGTTGAGC
l-87	AAGACTATGTTTAGTTCCAATCGTCAG	TCACTGGTGACTGAACAAGAAGTC
pre-c-HA87	GCCAGGGCTACACTGAGAAACTC	AGCGTAATCTGGAACATCGTATGGGTA
c-HA87	AAGACTATGTTTAGTTCCAATCGTCAG	AGCGTAATCTGGAACATCGTATGGGTA
pre-c-HA03	TTGGGCAGGTTTTATGTTTTGGGT	AGCGTAATCTGGAACATCGTATGGGTA
c-HA03	CAAGCTGGACCGCAGTGATG	AGCGTAATCTGGAACATCGTATGGGTA
Neomycin	CTTGGGTGGAGAGGCTATTC	TCAGTGACAACGTCGAGCAC
Atp5o	CAACCGCCCTGTACTCTGCT	GGATTCAGAACAGCCAGAGACAC
Bhlhe22	GCTGGTTGATGGGTCCGGAA	GCCTCTGAGTCCAATCCCGC
Chat	TGGAGAGACAGGAGAAGACAG	GCAGGGCTAGAGTTGACTGG
Gfap	GGCCCTGAGAGAGATTCGC	GCGTCTGTGAGGTCTGCAA
Hb9	TGCCAGCACCTTCCAACT	CTTCCCCAAGAGGTTCGACT
Isl1	TGTGGACATTACTCCCTCTTACA	TCGTGAATTTGATTGCCGCA
Olig2	GTTCTCCTCCGCAGCGAG	CTGGCGTCCGAGTCCATG
Pax6	GCCAGCAACACTCCTAGTCA	GTCTGTTCGGCCCAACATG
Pdgfrα	ACAACCACACTCAGACGGAT	TGACTAAGGAATCGGTCATCCC
Sim1	TGTCTCCCTTTGATGGATGCT	GCGTAGGTGGAAGGTGTCAC

Primers used in ClIP experiments for RT-PCR analysis in mouse samples:

pre-FUS	ATTTGGTGGTAAGTGAACAGAGTTT	CCCTTCTGGAGGTGGCTACA
pre-Atp5o	TGGAACTTACAGCAACACACGC	CTCAGTGACAATGCCATCCCTA
c-03 5'	CATCTTGGTTGGTCAGTCTGTGTG	CACCCTTGGGCATCGTTCTC
c-03 3'	CAAGCTGGACCGCAGTGATG	TGCTGTCTCTTGCCCTTACTGTT
c-03 NEG	GGGATGAGAACAAGTCTGTGCG	GGTTGTGACCTCTCGGGGA
c-13 5'	AAGAAGTAGAGTTTTAAGAATAGGCG	CTTTTCCAATTTGATGTAGGTTTCC
c-13 3'	TTGCCCGAGAGCGTATCAAT	GTGAAATGGTTTATGACAAACACAC
c-13 NEG	CCTAGAGTGATATTTTTCTTCAGCAA	CAGTCTCCTCTTAAACTTTTTCATCC
c-27 5'	TCTTATTCCCACATTGTAATTGAAGT	TCCAAAAAGATGTGTCACAAGCC
c-27 3'	CTTCACCCACCCTTCCTTCG	AAAATGGAGTCACATGAGCATACAG
c-27 NEG	GGATTAGAAAGATTGGCGATGG	TTTCAAGTAGTGGAACCTCCTAGATT
c-45 5'	TTGGATCTTCGCACTAACATCTG	CCTCATCCTCTTCTTCATCAGTGG
c-45 3'	GGAGACACTATGGAGAATGTGGAAG	GCTCTTTGCCTTGACACTCAT
c-45 NEG	GAAAAGCGAGTAGAAAAACTGGAAC	TCTACCCTCTGGAAAAGTCAATAATGT
c-75 5'	TTATCTGGTGGAAAGTTTGGTTG	GCATACTCTAACATCTTTATTCTTCTCAC
c-75 3'	CACTGGTAGTGCGGTAATTTATGAT	TCCTCCTTCATGTAATAATAATCTTGTA
c-75 NEG	ATGGTGATTGTTTTATGTTGCCTC	ACTTGTAACAGCATCCAAATGAGC
c-87 5'	TGGGCTTTTCTCTACAAATATCAAC	TACTCTGATTTTACACGCTTCCG
c-87 3'	TTGGAAAGAACTGAAACCTTAAACC	GCCACTGATACACACTGAAACAAG
c-87 NEG	TACTGCTGGCACCGTCTGATG	GCTGCTACTATAAACAAGTCAACTGG

Primers used for RT-PCR analysis in human samples:

hs-c-01	TCTCCAACGACCTGAAACGG	GGCTGTTCACGAAGGCATCG
hs-c-03	GAAGGACTCGGACACAGACGG	CCCAGTCTGCTTCTCCACCAT
hs-c-16	GTCAGACTTCGTAAGAGAAAGGTGG	GTCATCGTAGTTTAACAACAGACAGAGA
hs-c-27	CCTTTTACACATCCTTCCTTTGAGA	CCTACCTTTATACCAGCAAATAACTGA
hs-c-31	TCCAGGAGATGAAGATGACAAAGAC	CTTTGGGACCTAGAAATGCAGTT
hs-c-45	CCAAGATGGCCTAAGTCAAGACA	CTTGCTTGTGTCCAGTTCTGATG
hs-c-48	GCACCAGTGGCAAAAAGCG	CTCTCCTCTATATCCATAGTACCCGT
hs-c-52	CTTAACTGGAGCAACTGAGGGTG	TCCTGGGTTTACAGAAGTCATTCA
hs-c-75	ACTTTCATCACAGGTAGATTCTGGC	TCTTCTTACTAAGTCCTTCTTCAGGTTC
hs-c-78	TTTCTTCGTACTGGACAGTTTAGACTT	GGGCAATCTTCTAACAAGGCAT
hs-c-79	AGGATGTTGATGATGAGGACCA	ATTCTGTGCGATGCTGTCTGG
hs-c-80	AGAAGTTCACAGAAAACACTCCAAG	CCATCTTCTTTACAGACTCCTCCATA
hs-c-82	CCACCAGAGGACTCACACAGACAT	GGGTCTAAGCGACGGAAGC
hs-c-83	TCAAGCATTGGAAGAATTTAGGG	TTGTCCAAAGGAATTCACAGATCA
hs-c-84	CGAGGACTCTGAAAAAGGGGC	GCTGTGGTGGTGCTAAGGAATC
hs-c-87	AAGAGTATGTTTAGTTCCAATCGTCAG	CAGTCTGGCCCATGATTATTAGAAG
hs-c-88	CCGAGAACTGGAAGAGATGGG	GGCTCAGTATCTTAGGCAATGTCTAG
ATP5O	ACTCGGGTTTGACCTACAGC	GGTACTGAAGCATCGCACCT

Primer used for RT-qPCR analysis in human samples:

hs-c-80	AGAAGTTCACAGAAAACACTCCAAG	CCATCTTCTTTACAGACTCCTCCATA
hs-c-84	CGAGGACTCTGAAAAAGGGGC	GCTGTGGTGGTGCTAAGGAATC
ATP5O	ACTCGGGTTTGACCTACAGC	GGTACTGAAGCATCGCACCT
ChAT	TCATTAATTTCCGCCGTCTC	GAGTCCCGGTTGGTGGAGT
HB9	GAGACCCAGGTGAAGATTTG	CCTTCTGTTTCTCCGCTTCC
ISLET1	TACAAAGTTACCAGCCACC	GGAAGTTGAGAGGACATTGA
NANOG	CCAAATTCTCCTGCCAGTGAC	CACGTGGTTTCCAAACAAGAAA

Primer used for probes amplification in Northern Blot analysis:

T7-c-31	TAATACGACTCACTATAGGGATAGGGTACTTGTTTGCTGGAGG	AGACGCTGGCAATGACACGA
T7-c-78	TAATACGACTCACTATAGGGTAAGATATCATCTGCCAAACTGAG	CATTGAATATAGACCTGAAAACCATC

Primer used for circRNA cloning:

c-87 intron1-exon2	GTTTAAACTTAAGCTTTCTCTGTGTAGTCTTGGCTGTCTTG	CTTTACTTCATCAGCTCTTCTGAACC
c-87 exon3-intron3	GCTGATGAAGTAAAGACTATGTTTAGTTCCAATCGTCAGA	CTGGACTAGTGGATCCTCCACAAGGACAAAATCTGAACTC
c-87 HA tag insertion	GTTCCAGATTACGCTAATCATGGGCCAGACTGGGA	ATCGTATGGGTAATTCTAAAAGCAATGATTTCATATTATAATCACTT
c-03 intron1-exon2	TACCGAGCTCGGATCCCGGAGCCTTGAGGTTTAGAAGCAG	ACTTTATCTTTATCCTTTTTCCCAGTTTGTTTCTCCACCA
c-03 exon3-intron3	AAAGGATAAAGATAAAGTTTCTCTAACCAAG	GCCCTCTAGACTCGAGTCCACTCCATCATACCCCGT
c-03 HA tag insertion	TCCAGATTACGCTAAGCAATAGCAGCATAAATGAAGAT	ACATCGTATGGGTACATCATCAACTGTCTGTTTCAAA
